# Clinically relevant preclinical animal models for testing novel cranio‐maxillofacial bone 3D‐printed biomaterials

**DOI:** 10.1002/ctm2.690

**Published:** 2022-02-15

**Authors:** Luan P. Hatt, Keith Thompson, Jill A. Helms, Martin J. Stoddart, Angela R. Armiento

**Affiliations:** ^1^ Regenerative Orthopaedics Program AO Research Institute Davos Davos, Platz Switzerland; ^2^ Department of Health Sciences and Techonology Institute for Biomechanics ETH Zürich Zürich Switzerland; ^3^ Division of Plastic and Reconstructive Surgery Department of Surgery, Stanford School of Medicine Stanford University Palo Alto California

**Keywords:** animal models, bone, calvaria, CMF, mandibular defect, orbital floor, tissue engineering, translational medicine

## Abstract

Bone tissue engineering is a rapidly developing field with potential for the regeneration of craniomaxillofacial (CMF) bones, with 3D printing being a suitable fabrication tool for patient‐specific implants. The CMF region includes a variety of different bones with distinct functions. The clinical implementation of tissue engineering concepts is currently poor, likely due to multiple reasons including the complexity of the CMF anatomy and biology, and the limited relevance of the currently used preclinical models. The ‘recapitulation of a human disease’ is a core requisite of preclinical animal models, but this aspect is often neglected, with a vast majority of studies failing to identify the specific clinical indication they are targeting and/or the rationale for choosing one animal model over another. Currently, there are no suitable guidelines that propose the most appropriate animal model to address a specific CMF pathology and no standards are established to test the efficacy of biomaterials or tissue engineered constructs in the CMF field. This review reports the current clinical scenario of CMF reconstruction, then discusses the numerous limitations of currently used preclinical animal models employed for validating 3D‐printed tissue engineered constructs and the need to reduce animal work that does not address a specific clinical question. We will highlight critical research aspects to consider, to pave a clinically driven path for the development of new tissue engineered materials for CMF reconstruction.

## Graphical abstract

Reconstruction of bone defects of the CMF region is a major and difficult surgical intervention. 3D printing is an ideal biofabrication tool to create patient‐specific tissue engineered bone scaffolds. An appropriate pre‐clinical animal model should be based on the targeted clinical indication. Implementing standardised guidelines for preclinical studies can improve translation.

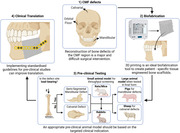

## 1 INTRODUCTION

Reconstruction of bone defects of the cranio‐maxillofacial (CMF) region, such as large segmental mandibular defects resulting from trauma, tumour excision, infections or congenital deformities, is a major surgical intervention. To date, transplantation of an autologous bone graft is the standard of care (SOC) to restore both the functional and aesthetic aspects of such defects.^1^ However, autologous bone grafting is associated with a number of important drawbacks including limited availability, donor site morbidity,^2^ a loss of osteogenic potential as the patient ages and, perhaps most importantly, the fact that autografts tend to undergo significant resorption over time. Together, these drawbacks highlight an urgent clinical need for alternative effective approaches to optimally restore bone tissue in the CMF arena.

To replace autologous bone grafts as the SOC, the replacement should demonstrate equivalent or improved functional and aesthetic outcomes, with minimal drawbacks. The bone graft substitute (BGS) should be osteoconductive, as well as osteoinductive, unlimited in supply, and mouldable to adapt to the irregular geometries frequently encountered in CMF reconstructions. Lastly, the material should be long lasting, that is, withstand resorption, and should integrate seamlessly into the existing bone tissue at a rate equivalent to that of an autologous bone graft. To date, no BGS has fulfilled all these functions. Although promising tissue engineering concepts have passed both in vitro and in vivo safety assessments,^3^ the BGS must also demonstrate superior performance, to effectively replace the current SOC and ensure wide‐scale clinical deployment. This requires robust preclinical models that closely recapitulate the features of the corresponding human clinical disease. In reality, the ‘recapitulation’ aspect has often been neglected, with many research groups failing to describe the clinical indication they are targeting, and/or the particular rationale for choosing a specific animal model.^4^ Without suitable guidelines that indicate the specific pathology being addressed, and a rigorous analysis of the most appropriate animal model, it should come as no surprise that most BGS fail to achieve the effects observed in preclinical studies when deployed in the clinical setting.

Although many technologies and fabrication innovations have been applied to produce such a BGS, the focus of this review concerns the use of the 3D printing approach, which is a particularly interesting tool for the CMF area since it allows for recapitulation of complex architecture and patient‐specific geometry. Given this focus, we discuss only 3D‐printed strategies used in preclinical studies as representative examples, to narrow the otherwise very large pool of publications in the field of bone tissue engineering.

This review reports the current clinical scenario of CMF reconstruction to first discuss the limitations of current preclinical models, followed by the ethical need to reduce animal work that does not address a specific clinical question and highlight critical research and clinical aspects. Factors to consider in choosing a preclinical model – including the anatomical location and type/size of the defect, as well as the incorporation of critical variables that affect patient outcomes, such as age and other co‐morbidities that potentially impact bone healing, are also discussed. To conclude, we propose a clinically driven path for the development of new tissue engineered BGS for CMF reconstruction.

## 2 CMF BONE STRUCTURE AND HEALING

**FIGURE 1:**
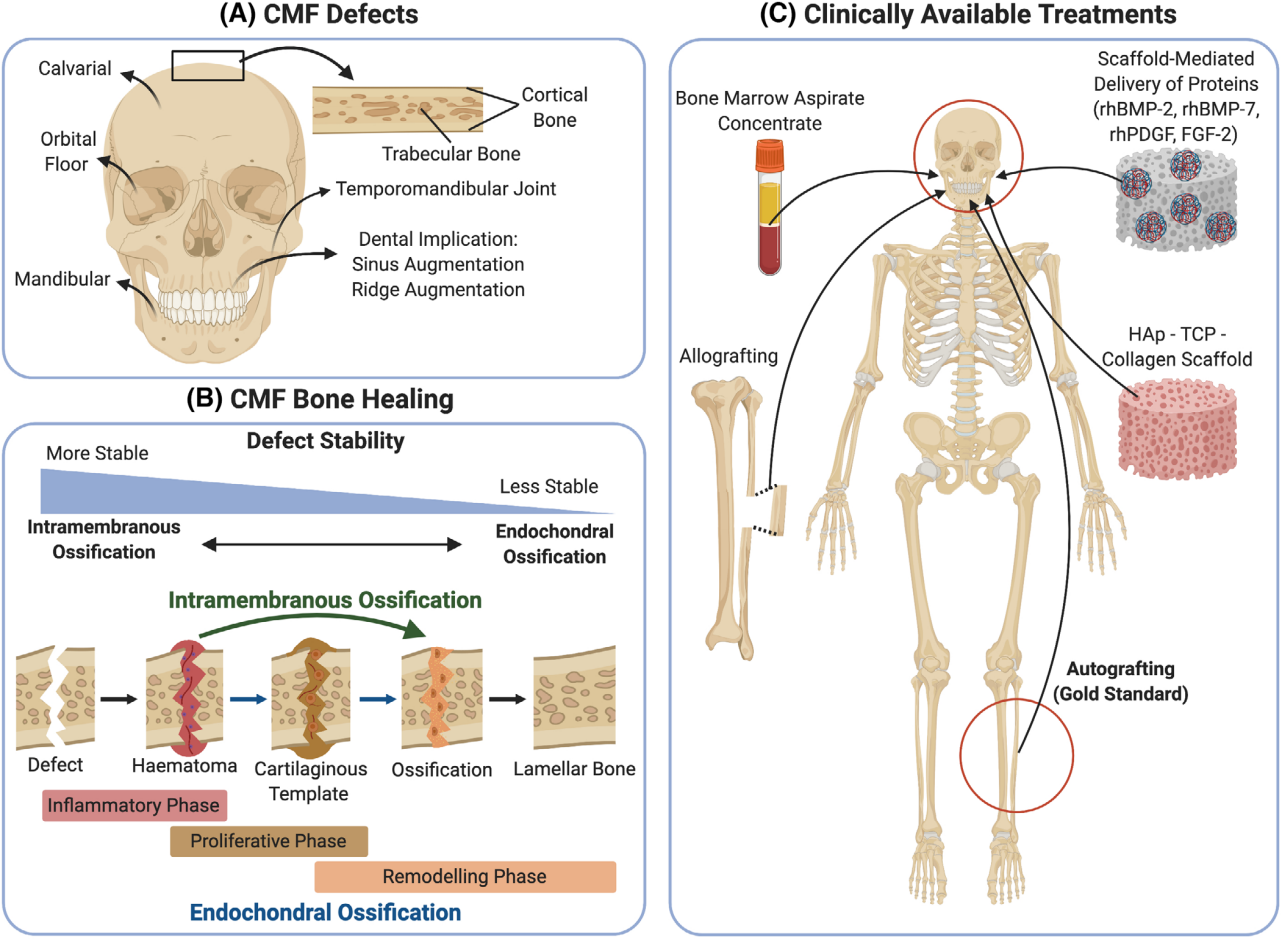
Schematic overview of (A) craniomaxillofacial (CMF) defects, (B) CMF bone healing through either intramembranous or endochondral ossfication and (C) clinically available treatments. rhBMP: recombinant human bone morphogenic protein; rhPDGF: recombinant human platelet‐derived growth factor; FGF‐2: fibroblast growth factor 2; HAp: hydroxyapatite; TCP: tricalcium phosphate. Created with BioRender.com

CMF bones (Figure 1A) not only differ in their healing process,^5^ but also differ in their structural framework and function. The macrostructure of CMF bone exists in the form of compact bone, which is permeated by interconnected canals called the haversian system, and cancellous bone, which has a porous structure that gives a honeycomb appearance. The interconnected haversian canals allow for a highly vascularised and innervated bone tissue. In the CMF complex, the bone supporting the teeth has a cancellous microstructure until teeth are lost then, concomitant with the edentulous state, cancellous bone is replaced by compact bone.^6^

Bone is a highly dynamic tissue that maintains its homeostasis through the process of bone remodelling. During this process, the activity of osteoblasts, osteocytes and osteoclasts is orchestrated by a multitude of tightly regulated molecular signalling pathways including canonical Wnt/β‐catenin and receptor activator of nuclear factor‐κB (RANK)/RANK ligand pathways. In the context of the CMF skeleton, osteoblasts have a dual origin, arising from either mesoderm (parietal and occipital bones) or the neural crest (frontal, ethmoid, sphenoid and facial bones),^7^, ^8^ with neural crest derived osteoblasts possessing a reported greater osteogenic potential.^9^

The regenerative capacity of CMF bone healing is often diminished (Figure 1B), perhaps in part due to limiting factors such as the relatively thin nature of the periosteum and the comparative lack of marrow space. A temporal and spatial coordinated response of numerous cell types is also required during bone healing.^10^ During the initial acute inflammation of bone healing, inflammatory cells including lymphocytes, macrophages, eosinophils and neutrophils are recruited to the haematoma of the fracture site.^11^ Important pro‐inflammatory cytokines in this process include TNFα, the interleukins IL‐1 and IL‐6, as well as growth factors such as fibroblast growth factor (FGF), platelet‐derived growth factor (PDGF) and transforming growth factor β (TGFβ), which initiate and coordinate the repair process.^12^, ^13^ The repair also involves vasculogenic and angiogenic responses driven by vascular endothelial growth factor (VEGF), and the recruitment of reparative progenitor cells including mesenchymal stromal cells (MSCs).^11^ These MSCs may then differentiate to either osteoblasts or chondrocytes, depending on the nature of the injury and the local mechanical environment, leading to the initiation of bone formation.^14^

Bone healing occurs via two distinct processes, termed endochondral or intramembranous ossification, which is critically dependent on the stability of the injured bone and the degree of interfragmentary strain generated during the reparative process.^15^, ^16^ The endochondral route of bone healing predominantly occurs in response to instability of the bone fragments, and is the major route of healing in long bones and vertebrae,^17^ as well as flat and irregular CMF bones without rigid stabilisation,^18^ as demonstrated by the presence of a cartilaginous tissue during the healing of a mandibular defect in a rabbit model^19^ and in a mouse mandibular fracture model.^20^

Intramembranous ossification is characterised by the direct differentiation of osteoprogenitor cells into osteoblasts^10^ and is the primary route for the formation of the flat bones in the cranium and some irregular bones such as the mandibles. To achieve direct ossification during bone healing, a correct anatomical reduction and a rigid fixation is required to limit movement of the bone fragments.^21^ Both bone‐healing mechanisms are tightly connected to angiogenesis and rely on the establishment of a functional vascularisation.^17^, ^22^

## 3 STANDARD OF CARE AND CLINICALLY AVAILABLE SOLUTIONS

Clinically available treatments for the reconstruction of bone defects are numerous and include autologous bone graft, allograft, demineralised bone matrix, hydroxyapatite (HAp), calcium phosphate, bone morphogenetic proteins (BMPs; e.g. BMP‐2 and BMP‐7), collagen scaffolds and bone marrow aspirate concentrate (Figure 1C).^23^

Autologous bone graft is the current SOC for bone reconstruction, with a 90% success rate, for which the free vascularised fibular flap was concluded to be a reliable source for the reconstruction of mandibular defects with positive aesthetic and functional outcomes including mastication, radiodensity of the bone and bone resorption rate, as well as minimum failure rate.^1^ Advantages of using an autologous bone graft are the availability of osteoprogenitor cells and the presence of a mineralised matrix scaffold that include desirable osteo‐inductive/conductive properties, thereby allowing the graft to integrate at the site of transplantation,^24^ and improved regeneration due to anastomosis of the vital tissue. However, numerous drawbacks of autologous bone graft use have been highlighted, including donor site morbidity issues (e.g. pain or infection);^25^ limited availability from the host;^26^ diminished osteogenic potential in older patients;^27^ significant loss in volume of the autologous bone graft over time due to resorption;^28^, ^29^ potential for increased blood loss due to extended surgical duration and lack of geometric accuracy for the defect site compared to its original shape.

Due to their increased availability, cadaveric donor allografts are also used clinically as an alternative to autologous bone grafts.^30^, ^31^ However, allografts are devoid of osteoprogenitors and pro‐osteogenic proteins^32^, ^33^ and have a rapid resorption rate,^34^ which diminishes their clinical efficacy.^35^

A tissue engineering alternative to autologous bone grafts is the scaffold‐mediated delivery of proteins such as rhBMP‐2,^36^ rhBMP‐7,^37^ recombinant human PDGF‐BB^38^, ^39^ or FGF‐2.^40^ Administration of rhBMP‐7 in combination with bovine collagen (OP‐1^®^), or rhBMP‐2 via a resorbable collagen sponge or HAp/β‐tricalcium phosphate (β‐TCP) (INFUSE^®^ Bone Graft and MASTERGRAFT™ Granules, respectively) are FDA‐approved options. INFUSE® Bone Graft is approved for lumbar spine fusion, open tibial fractures and CMF reconstruction and is often used off‐label for large segmental defects.^41^ However, the use of rhBMP has been associated with major side‐effects including ectopic bone formation,^42^ osteoclast‐mediated bone resorption,^43^ postoperative inflammation and inappropriate adipogenesis,^44^ as well as increased cancer risk for off‐label^45^ or high dosage rhBMP‐2 administration.^46^ Given these major safety issues, an FDA black box warning on high‐dose BMP‐2 was issued in 2008.^36^ Although BMP‐2 use has been associated with potential carcinogenic effects, this remains a contentious issue in clinical practice due to a limited number of high‐quality clinical studies on the subject.^47^ However, since CMF bone reconstruction is frequently required following tumour resection, further high‐quality and independent clinical studies involving the safe use of BMP‐2 are clearly warranted.

Thus, although autologous bone grafting remains the SOC in the clinic today due to the inherent limitations associated with its availability there is an urgent and, as yet, unmet clinical need for safe and viable alternatives.

## 4 TISSUE ENGINEERING AS A PROMISING ALTERNATIVE

Tissue engineering is a rapidly developing field that has been applied in multiple research disciplines, including the musculoskeletal realm. Tissue engineered constructs for bone applications are typically composed of a biocompatible, resorbable material with specific architecture, often combined with progenitor or differentiated cells and/or osteogenic/angiogenic growth factors to provide the biomolecular cues that mimic the complexity of bone tissue. While biological factors are crucial for the initial host cell invasion of the tissue engineered construct, it is evident that mechanical stability and porosity of the material play also an important functional role. A tissue‐engineered product targeting the restoration of a large bone defect must act as a place holder, promote the ingrowth of native tissue, and degrade over a suitable timeframe to allow regeneration of the defect area.

Modern tissue engineering increasingly relies on biofabrication technologies to design and manufacture complex biomimetic materials. Of particular interest for the regeneration of CMF defects is the process of additive manufacturing, also known as 3D printing, which allows the creation of complex patient‐specific solutions. 3D printing now refers to more than merely thermal‐based extrusion of polymers. The choice of material ranges from materials such as calcium phosphate pastes or metals to hydrogels and may also include cells, referred to as bioprinting. The use of 3D printing for CMF bone repair is summarised in a recent systematic review, which includes human and animal studies, with a focus on the scaffold's fabrication process and properties, as well as its combination with growth factors and cells.^48^ Different types of 3D printing technologies are also described, which encompasses inkjet printing, laser‐assisted printing and extrusion‐based printing.^48^ In further developments of 3D printing, novel 4D printing materials can be produced, which can change shape through the application of an external stimulus post‐printing.^49^

Commonly used materials in bone tissue engineering can be divided into calcium phosphate based materials, such as β‐TCP, HAp or calcium phosphate cement,^50^ and polymer‐based materials, such as polylactic acid, poly(lactic‐co‐glycolic acid: PLGA) and polycaprolactone (PCL),^51^ which are often combined to obtain an osteo‐conductive and/or ‐inductive engineered material. Several advanced approaches have been undertaken to improve material properties such as implementing piezoelectric materials, for example poly(vinylidene fluoridetrifluoroethylene) tested in the rat calvarial defect model^52^; incorporating magnetic components, for example magnetite nanoparticles into a nano‐HAp/chitosan/collagen mixture, tested in a rat calvarial defect model^53^; or exploiting further advanced strategies like biomimetic 4D printing^54^ or shape memory polymers, evaluated in a mouse femoral defect model,^55^ to name but a few examples. Additional advanced and smart biomaterials, and strategies to improve bone healing, have been covered in detail in numerous reviews.^3^, ^56^

Encapsulation of MSCs (derived from bone marrow, adipose tissue, or perivascular MSCs^57^) within the scaffold is another approach to improve the osteo‐conductive and ‐inductive properties of the construct by exploiting the secretome of the embedded MSCs, which has been shown to successfully heal a mouse calvarial defect when osteogenically pre‐differentiated MSCs were implanted in combination with a chitosan collagen microtissue,^58^ as well a rat calvarial defect with a dental pulp stem cell‐laden collagen gel scaffold.^59^ Incorporation of endothelial cells in co‐cultures with MSCs has also been used to create pre‐vascularised constructs to improve nutrient delivery, as shown by incorporating peripheral blood‐derived MSCs in combination with endothelial colony‐forming cells intro a PLGA/fibrin construct.^60^ Further strategies have been developed to create such a vascularised implant, for example, by perfusing HUVECs through a channelled biomaterial to create functional vessels,^61^ or via applying a flow bioreactor to a HUVEC‐laden biomaterial.^62^ Osteogenic factors can be chemically bound to the scaffold (biofunctionalisation), or the scaffold may be used as a carrier to deliver osteogenic and/or angiogenic factors, as demonstrated by creating a HAp complexed with BMP‐2 and VEGF peptides, tested in a diabetic rat femoral model.^63^ Increased delivery of these factors can be achieved by genetically engineering cells via gene delivery, as reported for hBM‐MSCs transfected with hBMP‐2 using a lentiviral vector system implanted into a intramuscular mouse model,^64^ and the implementation of hBM‐MSCs co‐transfected with hBMP‐2 and FGF‐2 via a polyethylenimine complex into a rabbit radial defect model.^65^

It is increasingly realised that the immune system plays a crucial role in the efficacy of tissue regenerative approaches. As such, the material of choice should prevent undesirable inflammatory responses and preferably promote favourable reparative immunological responses, such as the induction of reparative macrophage populations capable of secreting cytokines, TGFβ and interleukin 10, which have been shown to enhance bone formation.^66^ Furthermore, a specific population of periosteum‐resident macrophages (osteomacs) have been shown to play critical roles in bone homeostasis and the healing response following fracture.^67^ Thus, it is likely that successful bone reparative responses will require consideration of effects on such cell populations.

In addition, extracellular stimuli such as the stiffness, roughness and porosity of the material can also affect cell proliferation, migration and differentiation. Increased surface roughness of the material results in enhanced protein adsorption, thus improving cell attachment^68^ and osteogenic differentiation of MSCs.^69^ Effective invasion of cells is also dependent on the pore size of the material, as demonstrated in calcium‐based ceramic materials within which bone and blood vessel ingrowth requires a minimum pore size of 150 μm, with 400 μm being the upper limit for vascularisation.^70^ Material stiffness also affects cell morphology and differentiation. Fibroblasts acquire a round morphology on soft materials (180 Pa) while they flatten on stiffer ones (16 kPa),^71^ with similar effects observed with MSCs.^72^ Materials with a stiffer elastic modulus (25–40 kPa) also induce osteogenic differentiation of MSCs when compared to softer materials.^73^ Optimal biomaterial design strategies have been discussed by Dewey and Harley with specific insights on the importance of immune responses, as well as the interaction of multiple cell types for CMF bone healing.^74^

## 5 FROM A PROMISING TISSUE ENGINEERING CONCEPT TO A CLINICALLY JUSTIFIED PRODUCT

In vitro studies have identified a variety of promising materials of differing degrees of complexity and yet, with the exception of a scaffold‐based delivery of factors previously mentioned, most of the tissue‐engineered products identified to date have failed to demonstrate equivalent efficacy when compared to autologous bone grafts.

It is therefore important to consider the typical routine behind the development of a new material and discuss the reasons why the multitude of promising materials fail to translate into the clinical setting. Currently, the most widely used model for assessing material efficacy is the calvarial defect model in rodents. However, the specific features of the calvaria raise the question as to the wider applicability of findings from efficacy testing for other sites in not only the CMF region but also other parts of the skeleton, with their specific anatomical and mechanical loading environments. Thus, it is important to tailor a specific animal model according to the bone tissue of interest, to more faithfully recapitulate the appropriate clinical scenario in which the material will ultimately be deployed.

## 6 PRECLINICAL STUDIES TARGETING THE REGENERATION OF CMF BONE DEFECTS

In this section, we provide a series of recently reported applications of preclinical animal models to test the safety and efficacy of materials claimed to target CMF bone reconstruction. For clarity, the examples will be divided into calvarial, mandibular and orbital floor models. The presented studies are representatives from a large pool of preclinical studies using these models. Since 3D printing is a crucial technology for patient‐specificity, an inevitable direction for future CMF bone repair strategies, only studies that use a scaffold‐based tissue engineered approach fabricated using additive manufacturing printing have been included, with the aim to regenerate critical sized bone defects and using a CMF bone model. We did not focus on all biomaterials as these have been well described and discussed in other recent reviews.^74^, ^75^, ^76^ Further inclusion criteria are that the studies are being recent, being published between 2015 and 2021, and need to contain an animal study in which the 3D‐printed tissue‐engineered construct has been evaluated. Studies that involve 3D printing indirectly as a support system have also been excluded. Forty‐eight out of 75 papers were identified using PubMed for the calvarial defect model with the keywords ‘calvarial defect 3D printing’, 17 out of 123 for the mandibular defect model with the keywords ‘mandibular defect 3D printing’ and 1 out of 1 for the orbital floor defect model with the key words: ‘orbital floor defect additive manufacturing’. The search was conducted on the 8 October 2021. Multiple implant strategies (surface modification, drug and cell delivery) specifically aimed for bone tissue restoration were taken into consideration.

### 6.1 Calvarial defect model

Preclinical models involving calvarial bone defects are one of the most widely employed approaches to assess safety and efficacy of biomaterials due to their relative simplicity and reproducible nature. A sagittal incision is made to expose the calvarium and circular defects are created using a trephine burr.^77^ The anatomical location allows for easy surgical access and intraoperative handling without the need for internal or external fixation of the material.^78^ Reproducibility of the created defect and bone formation^79^ can be easily assessed radiologically and histologically^78^ but, due to the unloaded nature of the calvaria, the impact of mechanical stimulation cannot be routinely assessed in this model.^78^ Studies using the calvarial model to validate tissue engineered constructs are presented in Table 1. Common strategies include the delivery of drugs, biofunctionalisation of the construct, incorporation of MSCs, and the use of endothelial cells to create a prevascularised construct. Rats, followed by rabbits, are the most used preclinical animal models for this CMF region. The defects are generally created using a trephine burr and have a diameter ranging from 2.7 to 4 mm in mice, 4 to 9 mm in rats, 8 to 15 mm in rabbits and 11 mm in sheep, which are considered critical sized (and are therefore unable to heal naturally without intervention). The duration of the study typically ranges from 4 weeks up to 72 weeks, with time points typically starting after 2–4 weeks. Histological analysis (48 out of 48) is the prevailing method used to assess bone healing, together with CT scanning (35 out of 48) and immunohistochemistry (13 out of 48). Although the technique is relatively standardised and reproducible, care must be taken to avoid injuring the dura mater that leads to reduced healing.^80^ As previously mentioned, the periosteum is a critical tissue for bone repair, being a valuable source for regenerative cells, blood vessels for nutrient supply,^81^ but also contains sensory neurons. As a source of the neuropeptides substance P and calcitonin gene‐related peptide, sensory neurons appear to play important roles in the bone healing process,^82^ and fracture repair in long bones has recently been shown to require nerve growth factor expression in periosteal cells and tropomyosin receptor kinase A signalling in skeletal sensory nerve fibres.^83^ Therefore, damage to the periosteum may limit bone healing by interfering with these reparative processes and/or cell populations and should therefore be avoided where at all possible.

**TABLE 1:** Calvarial defect in preclinical animal models using the 3D printing approach

**3D‐printed material**	**Animal model[strain, age, weight, # groups (# defects per group)]**	**Defect(shape, size, number, # defects per animal, fixation)**	**Clinical aim**	**Animal model justification**	**In vivo methods (cutting tool, time points, analysis)**	**Results**
PCL/DCB scaffolds seeded with hASC^153^	Male FOXN1‐knockout mice, 8 weeks, 3 groups (*N* = 4)	Cylindrical 4 mm diameter, *n* = 1	**CMF bone regeneration**	No	4‐mm circular knife, 6, 12 weeks, CT, histology (H&E, Von Kossa/VGP)	Greater bone regeneration with PCL/DCP scaffolds compared to PCL only
CPC scaffold coated with BSP^155^	Female C57BL/6NRj mice, 6 weeks, 3 groups (*N* = 14)	Cylindrical 2.7 mm diameter, *n* = 1	CSD bone defect repair	No	Drill, 8 weeks μCT, histology (H&E, MGT), immunohistochemistry (OPN, PECAM‐1, vWF)	Enhanced bone repair with CPC/BSP compared to empty defect and tendency compared to CPC only (no significance)
β‐TCP/FA scaffold^154^	Female immunodeficient NOD.CB17‐Prkdc < scid > /J (‘NOD‐scid’) mice, 8–10 weeks old, 24 g, 5 groups, (*N* = 1 or 5) and C57BL/6JBomTac mice, 4 groups, (*N* = 2 or 6)	Cylindrical 4 mm diameter, *n* = 1	**CMF bone regeneration**	No	Biopsy punch, 8 weeks, μCT, histology (H&E), immunohistochemistry (Vimentin)	Sintered β‐TCP scaffolds visually promoted bone defect healing, but not β‐TCP/FA, but the groups did not show significant differences in quantification
C3S/MBG scaffold^119^	SD male rats, 12 weeks old, 250–300 g, 3 groups (*N* = 40)	Cylindrical 5 mm diameter, *n* = 2	Large bone defect repair	No	Dental trephine, 8 weeks, μCT, PSFL (2, 4 and 6 weeks), histology (VGP)	Improved osteogenic capacity of C3S/MBG compared to C3S only and empty defect
PLA/HAp scaffold^170^	SD male rats, 8 weeks old, 300–350 g, 4 groups (*N* = 8)	Cylindrical 5 mm diameter, *n* = 1	Large bone defect repair	No	Micro drills, 4 and 8 weeks, μCT, histology (H&E), immunohistochemistry (OCN, Col‐1)	Enhanced osteogenic capability with PLA/HAp compared to DBM and empty defect, but not to non‐printed β‐TCP
PCL/PLGA/β‐TCP scaffold doped with collagen^120^	SD male rats, 250–300 g, 2 groups (*N* = 16)	Cylindrical 8 mm diameter, *n* = 1	**Graft substitute for alveolar ridge repair**	No	Trephine burr, 2 and 8 weeks, histology (H&E, MT)	No enhanced bone regeneration with PCL/PLGA/β‐TCP compared to BCP
PCL scaffold coated with freeze dried PRP^121^	SD male rats, 200 g, 3 groups (*N* = 8)	Cylindrical 5 mm diameter, *n* = 1	Bone defect repair	No	Trephine burr, 2, 4, 8 and 12 weeks, μCT, histology (H&E)	Greater bone formation with freeze dried PRP/PCL compared to traditional PRP‐PCL and PCL only
CSH/MBG scaffold^122^	SD mature male rats, 250–300 g, 4 groups (*N* = 24)	Cylindrical 5 mm diameter, *n* = 2	Promote bone regeneration	No	Dental trephine, 8 weeks, μCT, histology (VGP)	Enhanced new bone formation with CSH/MBG compared to CSH only
PCL/β‐TCP/bdECM/BMP‐2 scaffold^123^	SD male rats, 12 weeks old, 250–300 g, 4 groups (*N* = 7)	Cylindrical 8 mm diameter, *n* = 1	Enhance bone repair	No	Trephine burr, 4 weeks μCT, histology (H&E)	Higher new bone volume and area with PCL/ β‐TCP/bdECM/BMP‐2 compared to PCL/β‐TCP/bdECM, PCL/β‐TCP and empty defect
β‐TCP scaffold with different pore sizes fabricated via 3D plotting^157^	SD rats, 12 weeks old, 180–200 g, 4 groups (*N* = 4–8)	Cylindrical 5 mm diameter, *n* = 2	Enhance bone repair	No	Drill, 4, 8 and 12 weeks μCT, mechanical testing, histology (H&E)	Highest stiffness and enhanced new bone ingrowth with β‐TCP (100 μm pore size) compared to 250 μm, 400 μm pore size and empty defect
PLA/PEG/nHAp/Dexa scaffold^124^	Female rats, 200–300 g, 3 groups (*N* = N/A)	Cylindrical 8 mm diameter, *n* = 1	CSD bone defect repair	No	Trephine burr, 4 and 12 weeks, μCT, histology (H&E)	Visually enhanced osteogenic response with PLA/PEG/nHAp with and without Dexa compared to empty defect
PIC/MWCNT scaffold^125^	SD male rats, 8 weeks old, 3 groups (*N* = 6)	Cylindrical 5 mm diameter, *n* = 1	Large bone defect repair	No	Trephine burr, 2, 4 and 8 weeks, μCT, histology (H&E, MT), immunofluorescence (Col‐1, Runx2, OCN) immunohistochemistry (CD31)	Promoted bone regeneration with PIC/MWCNT compared to PIC only and empty defect
HAp/F‐PLGA scaffold^126^	SD male rats, 500 g, 4 groups (*N* = 6–10)	Cylindrical 8 mm diameter, *n* = 1	**CMF bone regeneration**	No	Trephine burr, 8 and 12 weeks, CBCT, μCT, histology (H&E)	Enhanced bone repair with HAp/F‐PLGA compared to empty defect, but not to F‐PLGA only and autologous bone
AW/PLA (apatite‐wollastonite) scaffold Tcacencu^127^	SD male rats, 12 weeks old, 350 g, 3 groups (*N* = 3–6)	Cylindrical 8 mm diameter, *n* = 1	Mimic cortical and trabecular bone for better bone repair	No	Trephine burr, 12 weeks, histology (H&E, Wright‐Giemsa)	Enhanced newly formed bone with AW/PLA compared to AW only
HAp scaffold doped with BMP‐2^128^	SD male rats, 200–250 g, 3 groups (*N* = 8)	Cylindrical 5 mm diameter, *n* = 1	Bone defect repair	No	Trephine burr, 8 weeks, μCT, histology (H&E, MT)	Enhanced bone healing with HAp/BMP‐2 compared to HAp only and empty defect
PCL/PLGA/HAp scaffold combined with miR‐148b‐tranfected rat BM‐MSCs ^129^	Male Fischer 344 rats, 12 weeks old, 190–200 g, 3 groups (*N* = 5)	Cylindrical 5 mm diameter, *n* = 2	**CMF bone regeneration**	Yes	Trephine drill, 8 weeks, μCT, histology (H&E, MT), immunohistochemistry (DAPI, F‐ACTIN, Runx2, BSP)	More bone new formation with transfected cell‐laden scaffolds compared to cell‐laden scaffold without transfection and empty defect
BCP/CHAp granules or disks combined with TBM^130^	Lewis 1A‐haploype RT1a rats, 7 groups (*N* = 6)	Cylindrical 5.5 mm diameter, *n* = 2	**CMF bone regeneration**	Yes	Trephine burr, 7 weeks, μCT, histology (H&E, MGT), immunohistochemistry (CD31)	Greater new formation using BCP/CHAp/TBM disks compared to BCP/CHAp/TBM granules and empty defect
PEGDA/tECM scaffold fabricated via SLA^181^	SD rats, 4 weeks old, 3 Groups (*N* = 8)	Cylindrical 4 mm diameter, *n* = 2	Bone defect repair	No	N/A, 4 and 8 weeks, μCT, histology (H&E, MT, MGT)	Better bone defect repair with PEGDA/tECM compared to PEGDA only and empty defect
β‐TCP scaffold seeded with osteogenic‐ and angiogenic‐differentiated hUCMSCs^131^	SD rats, 12 weeks old, 250–300 g, 4 Groups (*N* = 6)	Cylindrical 5 mm diameter, *n* = 2	Large bone defect repair	No	Trephine burr, 4 weeks, μCT, histology (H&E, MT), immunohistochemistry (CD31, CD34)	Enhanced bone repair with osteo‐ and angio‐treated cell‐laden β‐TCP compared to osteo‐treated cell‐laden β‐TCP, β‐TCP only and empty defect
PLA/HAp scaffold seeded with BM‐MSCs with or without applying EMF^132^	SD male rats, 12–13 weeks old, 280–320 g, 5 Groups (*N* = 24)	Cylindrical 6 mm diameter, *n* = 1	**CMF bone regeneration**	No	Trephine burr, 4 and 12 weeks, μCT, μCT‐based micro angiography (6 weeks), histology (H&E, MT), mechanical testing	Higher new bone formation and improved neovascularisation with PLA/HAp/BM‐MSC/EMF compared to PLA/HAp/BM‐MSC, PLA/HAp/EMF, PLA/HAp and empty defect
Magnesium phosphate scaffold with and without micropores fabricated via salt leaching^133^	Aseptic male white rabbits, 12–15 weeks old, 4 groups (*N* = 10)	Cylindrical 4‐ and 6 mm diameter, *n* = 1	Study the effect of micropores for bone defect repair	No	Trephine burr, 4 and 8 weeks, μCT, histology (H&E, MT, TRAP)	Scaffolds with bigger micropores (25 and 53 μm) show better lamellar structure and enhanced calcification compared to no micropores and empty defect
Sr/MBG scaffold^147^	SD male rats, 12 weeks old, 3 groups (*N* = 6)	Cylindrical, 5 mm, *n* = 2	Bone defect repair	No	Trephine burr, 8 weeks, μCT, histology (tetracycline, alizarin red, calcein)	Enhanced new bone and vessel formation with Sr/MGB scaffold compared to MGB scaffold alone and empty defect
PCL coated with CaP or additional antimicrobial Se nanoparticles^182^	SD male rats, 12 weeks old, 2 groups (*N* = 6)	Cylindrical, 5 mm, *n* = 2	Preventing bacterial colonisation	No	N/A, 8 weeks, μCT, histology (H&E, MT)	Higher bone formation with PCL/CaP/Se scaffold compared to PCL/CaP
CDHAp/Col/BMP‐2 scaffold^148^	SD male rats, 8 weeks old, 240–260 g, 3 groups (*N* = 8–10)	Cylindrical, 8 mm, *n* = 1	**CMF bone regeneration**	No	Trephine burr, 8 weeks, μCT, histology (H&E)	Similar bone formation with CDHAp/Col scaffold and CDHAp/Col/BMP‐2 scaffold, but increased compared to empty defect
PCL scaffold functionalised with PRF^149^	SD male rats, 8 weeks old, 4 groups (*N* = 16)	Cylindrical, 6 mm, *n* = 2	**CMF bone regeneration**	No	Trephine burr, 4 and 8 weeks, radiography, μCT, histology (H&E)	Enhanced bone formation of scaffolds (PCL and PCL/PRF) compared to empty defect and PRF alone, but similar results between PCL compared to PCL/PRF scaffold
Methacrylate/silica scaffold^150^	SD male rats, 8 weeks old, 250 ± 15 g, 2 groups (*N* = 6)	Cylindrical, 8 mm, *n* = 1	Bone defect repair	No	Trephine burr, 8 and 16 weeks, μCT, histology (H&E, MT), immunohistochemistry (CD68, CD206, Col‐1, OCN, DAPI, vWF, α‐SMA)	Enhanced bone formation with methacrylate/silica scaffold compared to empty defect
PGSLP scaffold loaded with DFO‐laden gelatin nanofibers^183^	SD male rats, 4 weeks old, 6 groups (*N* = N/A)	Cylindrical, 5 mm, *n* = N/A	Bone defect repair	No	N/A, 6 and 12 weeks, μCT, histology (H&E, MT), immunohistochemistry (HIF1‐α, OPN, OCN)	Enhanced osteogenic and angiogenic activities with micro– and nanoporous structured PGSLP/DFO scaffold compared to empty control, PGSLP alone and porous structured PGSLP without DFO
β‐TCP scaffold coated with microRNA‐200c‐laden Col^169^	SD male rats, 12 weeks old, 6 groups (*N* = 5)	Cylindrical, 9 mm, *n* = 1	Bone defect repair	No	N/A, 4 weeks, μCT, histology (H&E, MT)	Enhanced bone regeneration with β‐TCP/Col/microRNA‐200c compared to empty defect β‐TCP alone, β‐TCP/Col and β‐TCP/microRNA‐200c
PCL scaffold combined with β‐TCP powder and/or dECM^174^	SD rats, N/A old, 4 groups (*N* = 3–8)	Cylindrical, 8 mm, *n* = 1	Treating bone fractures	No	Burr drill, 4 weeks, μCT, histology (MT), immunohistochemistry (myeloid‐related protein‐14 MRP‐14, OPN)	Faster bone formation and lower inflammatory response, with PCL/β‐TCP/dECM scaffold compared to PCL alone, PCL/β‐TCP and PCL/dECM
CSi/Mg scaffold^134^	NZ white rabbits, 2.8 kg, 4 groups (*N* = 16)	Cylindrical 8 mm diameter, *n* = 4	**Thin wall CMF bone defect repair**	No	Dental trephine burr, 6 and 12 weeks, mechanical testing, μCT, histology (VGP)	Enhanced new bone regeneration with CSi/Mg compared to CSi scaffolds only
CSi/Mg scaffold printed via SLP or DLP^135^	NZ male rabbits, 2.8 kg, 5 groups (*N* = ∼12)	Cylindrical 8 mm diameter, *n* = 4	**Thin wall CMF bone defect repair**	No	Dental trephine burr, 4, 8 and 12 weeks, μCT, histology (VGP)	Higher osteogenic capacity with DLP compared to SLP and enhanced bone repair with CSi compared to Csi/Mg and empty defect
PCL scaffold with different porosity^136^	NZ male rabbits, 12–13 weeks old, 3.4 kg, 4 Groups (*N* = 8)	Cylindrical 6 mm diameter, *n* = 4	**Graft substitute for dentistry**	No	Trephine burr, 4 weeks, μCT, histology (H&E)	Enhanced new bone formation in PCL with 30% porosity compared to 50%, 70% and empty defect
HAp scaffold coated with nanoparticles composed of BMP‐2 embedded in PCL^137^	NZ male white rabbits, 12 weeks old, 2–3 kg, 3 Groups (*N* = 4)	Cylindrical 6 mm diameter, *n* = 3	Bone defect repair	No	Trephine burr, 8 weeks μCT, histology (MGT)	Higher new bone formation with coated HAp compared to uncoated HAp and empty defect
PCL/β‐TCP/dECM scaffold^184^	NZ male white rabbits, 12 weeks old, 3–3.5 kg, 5 Groups (*N* = 3)	Cylindrical 8 mm diameter, *n* = 4	Large bone defect repair	No	Dental drill, 6 and 12 weeks μCT, histology (H&E, MT, Von Kossa)	Enhanced bone regeneration with PCL/β‐TCP/dECM compared to PCL/dECM, PCL/β‐TCP, PCL and empty defect
PCL scaffold^138^	NZ white rabbits, 12 weeks old, 2.5 kg, 2 groups (*N* = N/A)	8‐shaped, 5.6 and 7 mm diameter each defect (1 mm overlap, *n* = 2	Finding an optimal CSD model	No	Trephine burr, 1, 2, 4, 8, 12 and 16 weeks, CT, μCT, histology (H&E)	7 mm empty defect shows decreased bone healing abilities compared to 5 mm. Trend of enhanced bone repair with PCL compared to 7 mm empty defect (no significance)
Sr/HAp scaffold^158^	NZ male white rabbits, 3 Groups (*N* = N/A)	Cylindrical 15 mm diameter, *n* = 1	Enhanced bone augmentation and regeneration	No	Cranial drill, 4, 8 and 12 weeks, μCT, histology (H&E, MT)	Trend of more new bone formation with Sr/HAp compared to HAp only (no significance)
PCL/β‐TCP/Col scaffold^139^	NZ white rabbits, 2.8–3.2 kg, 4 groups (*N* = 10)	Cylindrical 8 mm diameter, *n* = 4	**Alveolar bone defect repair**	No	Trephine burr, 2 and 8 weeks, μCT, histology (MT)	Trend of more new bone volume with PCL/β‐TCP/Col compared to PCL/β‐TCP, PCL only and empty defect (no significance)
PGA scaffold combined with electrospun SF membrane^185^	Rabbits, 8 weeks old, 230–280 g, 3 groups (*N* = 3)	Cylindrical 8 mm diameter, *n* = 4	Bone defect repair	No	N/A, 4 and 8 weeks, μCT, histology (H&E, MT)	Trend of greater bone regeneration with PGA/SF compared to PGA only and empty defect (no significance)
PCL scaffold functionalised with BFP1^140^	NZ male white rabbits, 3–3.5 kg, 4 groups (*N* = N/A)	Cylindrical 10 mm diameter, *n* = 2	Enhance bone regeneration in dentistry and orthopaedic	No	Trephine drill, 8 weeks, radiography, histology (H&E, MT), immunohistochemistry (CD31, DAKO)	Higher stimulation of vessel and bone formation with PCL/BFP1 compared to PCL only
PCL scaffold using kagome structure vs. conventional grid‐type structure^141^	NZ white rabbits, 12–13 weeks old, 2.0–2.5 kg, 3 groups (*N* = N/A)	8‐shaped 7 mm diameter with 1 mm overlap, *n* = 2	**CMF bone regeneration**	No	Trephine drill, 4, 8, 12, 16 weeks, CT, μCT, histology (H&E, MT), immunohistochemistry (OCN)	Kagome structure shows improved mechanical robustness compared to grid type and enhanced bone repair compared to empty defect
BCP scaffold with different macro‐pore sizes^159^	NZ white male rabbits, 12 weeks old, 2.5 kg, 4 groups (*N* = 8)	Cylindrical 8 mm diameter, *n* = 4	Large bone defect repair	No	Drill, 4 and 8 weeks, μCT, histology (MGT),	Enhanced bone‐forming abilities with BCP scaffold compared to empty defect
β‐TCP scaffold doped with DIPY^142^	Skeletally immature NZ white rabbits, 4 weeks old, 3 groups (*N* = 8)	Cylindrical 10 mm diameter, *n* = 2	**Pediatric CMF bone reconstruction**	No	Trephine drill, 24 weeks, μCT, histology (SB, VGP)	Similar osteogenic regeneration with β‐TCP/DIPY compared to autologous bone
HAp scaffold coated with chitosan and sodium hyaluronate and loaded with BMP‐2 and VEGF^186^	NZ white rabbits, 2–3 kg, 3 groups (*N* = 12)	Cylindrical 15 mm diameter, *n* = 1	Bone defect repair	No	N/A, 4, 8 and 12 weeks, μCT, histology (H&E), immunohistochemistry (Col‐1, lectin)	Enhanced new bone formation with HAp/BMP‐2/VEGF compared to HAp only and empty defect
PCL/DCPD/nanoZIF‐8 scaffold^143^	NZ male rabbits, 2–3 kg, 4 groups (*N* = 6)	Cylindrical 10 mm diameter, *n* = 2	Bone defect repair	No	Trephine burr, 12 weeks, μCT, histology (H&E, MT), immunohistochemistry (OCN)	Enhanced new bone formation with PCL/DCPD/nanoZIF‐8 compared to PCL/DCPD, PCL only and empty defect
PCL functionalised with Bone graft scaffold^151^	NZ white male rabbits, 3–3.5 kg, 3 groups (*N* = 10)	Cylindrical 8 mm diameter, *n* = 3	**Guided alveolar bone regeneration**	No	Trephine burr, 2 and 8 weeks, μCT, histology (H&E, MT)	Increased new bone formation with PCL/Bone graft compared to empty defect and PCL alone
β‐TCP scaffold coated with DYPY^152^	NZ white male rabbits, 3–3.5 kg, 1 month old, 1 group (*N* = 21)	Cylindrical calvarial 10 mm; Squared alveolar 35 × 35 mm^2^ *n* = 2 (one defect each)	**CMF bone regeneration**	No	Trephine burr (calvarial), oral surgical burr (alveolar), 2, 6 and 18 months, μCT, histology (SB and VGP), mechanical testing	Similar bone percentage and mechanical properties with β‐TCP/DYPY scaffold compared to native bone
HAp/PLGA scaffold^160^	Rhesus macaque (non‐human primate), 11.65 kg, 1 group (*N* = 2) **(Case study)**	Square, 40 × 40 mm^2^ *n* = 2	Discover ideal bone graft material	Yes	Rongeurs to expand pre‐existing defects, 4 weeks, mechanical testing, μCT, histology (H&E)	New woven bone was observed
β‐TCP scaffold coated with DIPY^187^	Mature Dorset/Finn sheep, 62 kg, 2 groups (*N* = 10)	Cylindrical 11 mm diameter, *n* = 4	Enhanced bone regeneration	Yes	Trephine hurr, 3 and 6 weeks, histology (SB, VGP)	Higher bone formation with β‐TCP/DIPY compared to β‐TCP only

NA, data not available; PCL: polycaprolactone; DCB: decellularised bone; hASC: human adipose‐derived stem cell; CMF: cranio‐maxillofacial; CT: computed tomography; H&E: haematoxylin and eosin; VGP: Van Gieson's picrofuchsin; CPC: calcium phosphate cement; BSP: bone sialoprotein; CSD: critical‐sized defect; μCT: micro CT; MGT: Masson Goldner Trichrome; OPN: osteopontin; PECAM‐1: platelet endothelial cell adhesion molecule‐1; vWF: von Willebrand factor; β‐TCP: β‐tricalcium phosphate; FA: fatty acid; C3S: tricalcium silicate; MBG: mesoporous bioactive glass; SD: Sprague Dawley; PSFL: polychrome sequential fluorescent labelling; PLA: polylactic acid; HAp: hydroxyapatite; OCN: osteocalcin; Col‐1: collagen‐1; DBM: demineralised bone matrix, PLGA: poly(lactic‐co‐glycolic acid), MT: Masson's Trichrome; BCP: biphasic calcium phosphate; PRP: platelet‐rich plasma; CSH: calcium sulphate hydrate; bdECM: bone demineralised and decellularised extracellular matrix; BMP‐2: bone morphogenic protein‐2; PEG: polyethylene glycol; nHAp: nano HAp; Dexa: dexamethasone; PIC: polyion complex; MWCNT: multiwalled carbon nanotubes; Runx2: runt‐related transcription factor 2; F‐PLGA: fluffy PLGA; CBCT: cone beam CT; BM‐MSC: bone marrow‐derived mesenchymal stem cells; CHAp: carbonated HAp; TBM: total bone marrow; PEGDA: polyethylene glycol diacrylate; tECM: tendon ECM scaffold; SLA: stereolithography; hUCMSCs: human umbilical cord MSC; EMF: electromagnetic field; Sr: strontium; CaP: calcium phosphate; SE: selenium; CDHAp: calcium‐deficient hydroxyapatite; PRF: plasma‐rich‐fibrin; α‐SMA: alpha‐smooth muscle actin antibody; PGSLP: poly (glycerol‐co‐sebacic acid‐co‐L‐lactic acid‐co‐polyethylene glycol); DFO: deferoxamine; HIF1‐α: hypoxia inducible factor 1‐alpha; MRP‐14: myeloid‐related protein‐14; CSi/Mg: wollastonite doped with dilute magnesium; NZ: New Zealand; SLP: single‐layer printing; DLP: double‐layer printing; PGA: poly(glycolic acid); SF: silk fibroin; BFP1: bone forming peptide 1; DIPY: dipyridamole; SB: stevenel blue; VEGF: vascular endothelial growth factor; DCPD: dicalcium phosphate dihydrate; nanoZIF‐8: nanoscale zeolitic imidazolate framework‐8.

### 6.2 Mandibular defect model

**FIGURE 2:**
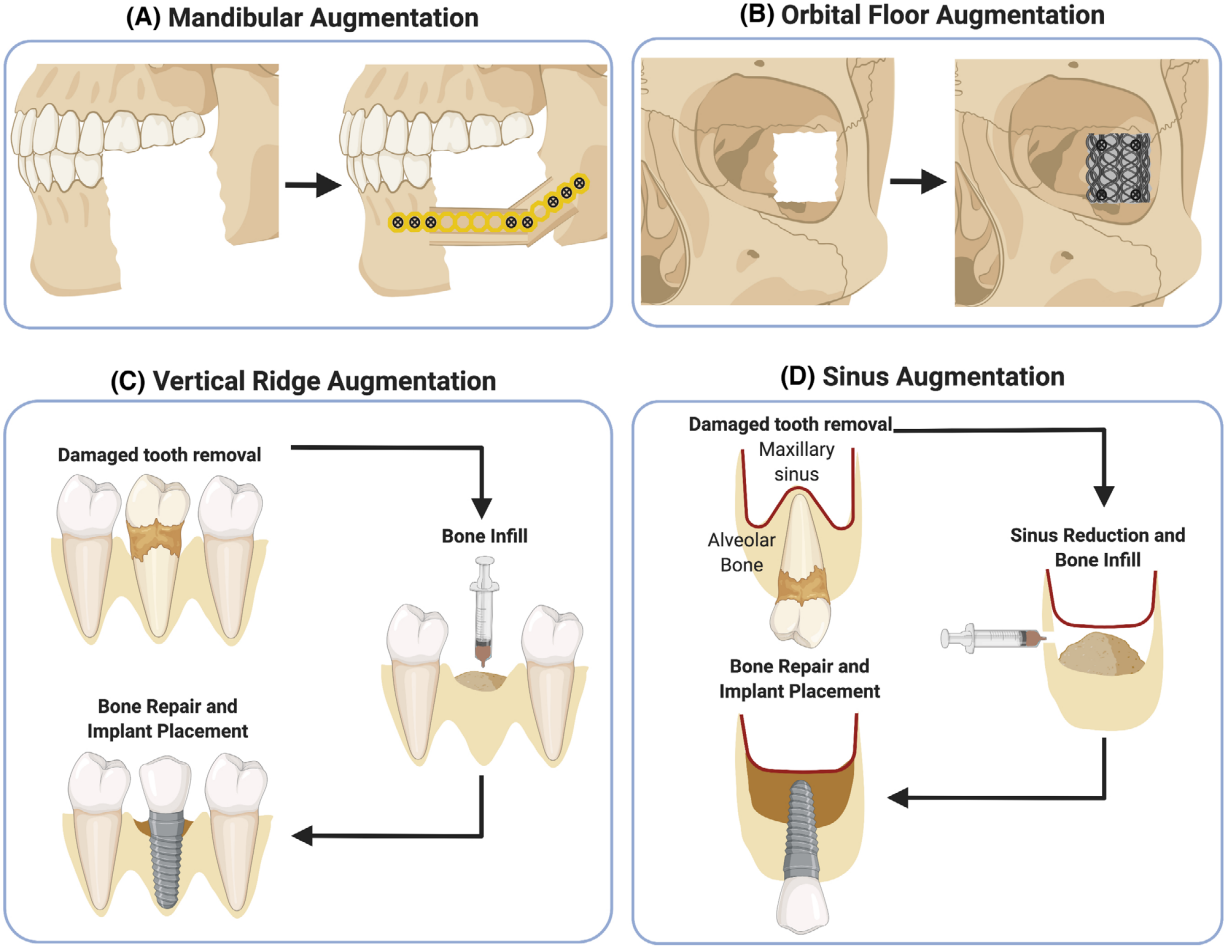
CMF augmentation techniques. Created with BioRender.com

Mandibular defects can be distinguished in continuity and non‐continuity defects. Non‐continuity defects have a circular or rectangular geometry without the loss of the mandibular unity so that an additional mechanical fixation is not required. These defects are more often used in small animal models that can provide information on both biocompatibility and efficacy of tested constructs, but often fail to adequately mimic the clinical setting such as load‐bearing and size, as previously described for calvarial defects.^84^ A clinical example for using non‐continuity defects in a preclinical model is bone healing following tooth extraction. Continuity defects are typically segmental resections with loss of mandibular continuity, such as those seen clinically following tumour resection, and therefore require internal fixation to provide adequate mechanical stability, illustrated in Figure 2A. Due to the complexity of the procedure, this type of defect is more often used in large animal models, with which the clinical condition is more accurately resembled^84^ and the load‐bearing capacities of BGSs can be adequately assessed.^78^

Preclinical studies using the mandibular defect model are shown in Table 2. In addition to small animal models, such as rats and rabbits, large animal models such as minipigs, beagle dogs and sheep are also used. Most of the created defects are semi‐ or completely segmental and are therefore created using a saw instead of a burr. Semi‐ or completely segmental defects have a high range of sizes used across different species, with 30 mm^3^ in rats, 240–750 mm^3^ in rabbits, 105–2000 mm^3^ in beagle dogs and 2800 to 12 000 mm^3^ in pigs. Typically, cylindrical defects are created using a burr and have a diameter of 4–5 mm in rats and 8 mm in rabbits. As described for the calvarial defect, histological‐based assessment (17 out of 17) is the main method to assess healing, and most studies also include CT scanning (15 out of 17) and some include immunohistochemistry (3 out of 17) or mechanical testing (2 out of 17) as additional approaches. Study duration is typically 8 or 12 weeks, with longer studies up to 24 and 32 weeks. Complications associated with mandibular defects include microbial infections when using intra‐oral approaches,^85^ and also plate failure in continuity defects.^86^ In addition, the choice of suitable animal species for mandibular defects is complicated due to confounding effects of the long, continuously erupting incisors present in small animal models (e.g. mice, rats, rabbits). Such mandibular defects typically cut into the tooth in such species, with resulting injury to the tooth, periodontal ligament, cementum, dentine and pulp. As such, these mandibular defects in small animals are markedly different to equivalent defects in large animal models and patients.

**TABLE 2:** Mandibular defect in preclinical animal models using using the 3D printing approach

**3D‐printed material**	**Animal model [strain, age, weight, # groups (# defects per group)]**	**Defect (shape, size, number, # defects per animal, fixation)**	**Clinical aim**	**Animal model justification**	**In vivo methods (cutting tool, time points, analysis)**	**Results**
HAp/PLGA scaffold^144^	SD male rats, 250–300 g, 2 groups (*N* = 6–8)	Cylindrical 4 mm (0.5 mm depth), *n* = 2	**Alveolar ridge augmentation**	No	Trephine burr, 4 weeks, early gene expression (day 7, Col‐A1, VEGF, Cbfa1), μCT, histology (H&E, MT)	Enhanced newly formed bone with HAp/PLGA compared to empty defect
PVA/PU ‘LayFomm’ scaffold^162^	SD male rats, 6–8 months old, 2 groups (*N* = 6)	Semi‐segmental 5 × 2 × 3 mm^3^ *n* = 2	**Craniofacial bone repair**	No	Spherical burr, 6 weeks, μCT, histology (ALP, TRAP)	Increased mineralised tissue production with LayFomm compared to Norian putty
PCL/TCP/ME‐HA/ME‐Gel scaffold fabricated via dual printing doped with RVS and SrRn^145^	SD male rats, 280–330 g, 3 groups (*N* = 5–6)	Cylindrical 4 mm, *n* = 2	**CMF bone reconstruction**	No	Trephine burr, 8 weeks, μCT, histology (H&E, MT)	Promoted bone formation with drug loaded scaffolds compared to scaffold only and empty defect
Ti6Al4V scaffold combined with ADSC‐laden Cellmatrix hydrogel^167^	SD rats, 8 weeks old, 3 groups (*N* = 3)	Cylindrical 5 mm (1 mm depth), *n* = 1	**Mandibular bone defect repair**	No	N/A, 12 weeks, μCT, histology (VGP)	Highest amount of new bone with Ti6Al4V/ADSC/Cellmatrix compared to Ti6Al4V/ADSC and Ti6Al4V only
PEKK scaffold fabricated via SLS seeded with ADSC^163^	NZ rabbits, 2 groups (*N* = 12)	Semi‐segmental trapezodial, 15 × 10 × 5 mm^3^ *n* = 2	**Craniofacial bone reconstruction**	No	Diamond burr, 10 and 20 weeks, μCT, mechanical testing, histology (MGT)	Enhanced bone repair with PEKK/ADSC compared to empty defect. Higher compressive resistance in PEKK/ADSC compared to PEKK only and bone
CSi/Mg scaffold^188^	NZ male rabbits, 4 groups (*N* = 16)	Square 10 × 6 × 4 mm^3^ *n* = N/A	**Alveolar bone repair**	No	N/A, 8 and 16 weeks, radiography, CT, histology (N/A)	Higher osteogenic capability with CSi/Mg compared to β‐TCP, CSi only and bredigite after 16 weeks
β‐TCP scaffold^189^	NZ adult rabbits, 35 kg, 1 group (*N* = 5) (Pilot Study)	Complete segmental, 11 × 9 × 4.5 mm^3^, *n* = 1, plate	**Mandibular bone reconstruction**	No	N/A, 8 weeks, μCT, histology (Stevenel's blue and VGP), backscatter electron microscopy	No enhanced bone repair with β‐TCP compared to native bone
PLGA/nHAp scaffold containing BMP‐2 and chitosan^168^	NZ white rabbits, 13 weeks old, 2.5–3.5 kg, 2 groups (*N* = 18)	Semi‐segmental 13 × 6 × 4 mm^3^ *n* = 2	**Large maxillary bone defect repair**	No	N/A, 4, 8 and 12 weeks, μCT, histology (H&E, MT), immunohistochemistry (OCN)	Greater bone repair with PLGA/nHAp/BMP‐2/chitosan compared to PLGA/nHAp
PCL/β‐TCP scaffold seeded with osteogenic pre‐differentiated TMSCs^164^	NZ male white rabbits, 12 weeks old, 2.5–3 kg, 5 groups (*N* = 2–4) **(Pilot Study)**	Semi‐segmental, 10 × 8 × 5 mm^3^ *n* = 1, fixing plate	Large bone defect repair	No	Surgical drill and osteotome, 12 weeks, CT, μCT, mechanical testing, histology (H&E, MT, alizarin red S), immunohistochemistry (CD31)	Trend of enhanced repair with PCL/β‐TCP/TMSCs (differentiated) compared to PCL/β‐TCP/TMSCs (undifferentiated), PCL/β‐TCP/PB, PCL/β‐TCP and empty defect (no significance)
Bioglass scaffold functionalised with boron^146^	NZ male rabbits, 3.5–4 kg, 3 groups (*N* = 4)	Cylindrical 8 mm (2 mm depth), *n* = 1	**Mandibular bone defect repair**	No	Trephine burr, CT (4 and 8 weeks), histology (2 and 4 weeks, H&E)	Enhanced bone repair with Bioglass/boron compared to HAp and empty defect
HAp/TCP scaffold fabricated via digital light processing‐type 3D printing^172^	Adult beagle dogs, 3 groups (*N* = 16)	Semi‐segmental 7 × 3 × 5 mm^3^ *n* = 2, fixation pins	**Alveolar ridge augmentation**	No	Dental burr, 4 and 8 weeks, μCT, histology (H&E, MGT)	No difference in new bone formation with HAp/TCP compared to OSTEON
PCL/β‐TCP doped with either BMP‐2 or ABP^161^	Male beagle dogs, 12–14 months old, 12.5 kg, 4 groups (*N* = 4)	Semi‐segmental 20 × 10 × 10 mm^3^ *n* = 2, fixation via wing structures on the scaffold	**Oromandibular bone reconstruction**	No	Reciprocating bone saw, 12 weeks, μCT, histology (H&E, MT)	More bone formation in drug‐doped scaffolds compared to non‐doped scaffold and empty defect
PCL/β‐TCP doped with rhBMP‐2^164^	Male beagle dogs, 12–14 kg, 4 groups (*N* = 4)	Semi‐segmental 10 × 5 × 5 mm^3^ *n* = 4,	**Mandibular bone defect repair**	No	Surgical burr, 12 weeks, μCT, histology (MT)	Increased newly formed bone with PCL/β‐TCP compared to PCL only with or without rhBMP2
OCP scaffold functionalised with pDNA encoding VEGFA^190^	Adult male pigs, 50 Kg, 2 groups (*N* = 4), titanium miniplate	Semi‐segmental 25 × 15 × 10 mm^3^ *n* = 2, surgical guide plate	Large bone defect repair	No	N/A, 3 and 6 months, CT, histology (H&E)	No enhanced bone repair with OCP/pDNA‐VEGFA compared to OCP only
β‐TCP/PCL scaffold seeded with osteo‐treated pBM progenitor cells^92^	Yucatan minipigs, N/A, 3 groups (*N* = 3–6), **(Pilot study)**	Semi‐segmental 20 × 20 × 7 mm^3^ *n* = 6	**Improving deep bone penetration for craniofacial reconstruction**	Yes	Reciprocating bone saw, 8 weeks, histology (H&E, DAPI), immunohistochemistry (CD31)	More bone formation with cell‐laden β‐TCP/PCL compared to cell‐free β‐TCP^/^PCL, but less in the empty defect.
PLA scaffold coated with poly‐electrolyte film to deliver BMP‐2^166^	Mature female minipigs, 24 months old, 43–69.5 kg, 4 groups (*N* = 4–6) titanium plates	Squared, 40 × 30 × 10 mm^3^	**Mandibular bone defect repair**	Yes	Oscillating saw, ca. 2, 4, 12 weeks and 3 months, CT, μCT, histology (VGP)	Enhanced bone formation compared empty defect and no significant difference to positive control
Fullcure scaffold fabricated by SLA^173^	‘Lataxa’ Asturian female sheep, 15 months old, 59 kg, 1 group **(Case Study)**	Complete segmental 30 mm, *N* = 1, osteosynthesis plate	**Evaluate bioactivity of Fullcure**	No	N/A, 32 weeks, CT, histology (H&E)	Fullcure did not guide and assure the shape of newly generated bone

NA: data not available; HAp: hydroxyapatite; PLGA: poly(lactic‐co‐glycolic acid); SD: Sprague Dawley; Col‐A1: collagen‐A1; VEGF: vascular endothelial growth factor; Cbfa1: core‐binding factor alpha‐1; μCT: micro computed tomography; H&E: haematoxylin and eosin; MT: Masson's Trichrome; PVA: polyvinyl alcohol; PU: polyurethan; ALP: alkaline phosphatase; TRAP: tartrate resistant acid phosphatase; PCL: polycaprolactone; TCP: tricalcium phosphate; ME‐HA: methacrylated hyaluronic acid; ME‐Gel: methacrylated gelatin; RVS: resveratrol; SrRn: strontium ranelate; CMF: cranio‐maxillofacial; Ti6Al4V: alpha beta titanium alloy; ADSC: adipose‐derived stem cell; VGP: Van Gieson's picrofuchsin; PEKK: polyetherketoneketone; SLS: selective laser sintering; NZ: New Zealand; MGT: Masson Goldner Trichrome; CSi/Mg: wollastonite substituted with magnesium; nHAp: nano hydroxyapatite; BMP‐2: bone morphogenic protein‐2; OCN: osteocalcin; TMSC: tonsil‐derived mesenchymal stem cell; ABP: autogenous bone particles; rhBMP‐2: recombinant human BMP‐2; OCP: octacalcium phosphate; pDNA: plasmid deoxyribonucleic acid; pBM: porcine bone marrow; SLA: stereolithography.

### 6.3 Orbital floor model

Common materials utilised to clinically reconstruct the orbital floor following injury include metal alloy, titanium, polylactic acid and HAp composites. These reconstruction strategies target the replacement rather than regeneration of bone, illustrated in Figure 2B. Only one preclinical study with the aim to regenerate the lost bone in the orbital floor was chosen according to the criteria and is presented in Table 3. The described sheep study involved an irregular shaped defect created using a retractor and pean forceps, being 6 × 9 mm^2^ in size.^87^ Histological analysis is, again, the main evaluation method used, in addition to CT scanning. Specific for this model, the restoration of the normal position of the eyeball within the socket is often assessed. The duration of the animal study is 12 weeks. Complications arising from the surgical approach in this model have not been reported.

**TABLE 3:** Orbital floor defect in preclinical animal models using the 3D printing approach

**Tissue engineering approach**	**Animal model [strain, age, weight, # groups (# defects per group)]**	**Defect (shape, size, number, # defects per animal, fixation)**	**Clinical aim**	**Animal model justification**	**In vivo methods (cutting tool, time points, analysis)**	**Results**
SLA 3D‐printed resorbable PTMC/HAp scaffold^87^	Female skeletally mature, Swiss White Alpine sheep, 2–4 years old, ∼69 kg, 2 groups (*N* = 6)	Irregular shape, 6 × 9 mm^2^, *n* = 1, fixation via titanium microscrews	**Repairing orbital floor defects**	Yes	Retractor + pean forceps, 4, 8, 12 weeks, CT, histology (Giemsa‐Eosin)	Higher bone formation of resorbable scaffold compared to standardly used titanium mesh

NA: data not available; SLA: stereolithography; PTMC: poly(trimethylene carbonate); HAp: hydroxyapatite; CT: computed tomography.

### 6.4 Vertical ridge augmentation and sinus augmentation

Additional CMF‐relevant issues include dental reconstructive approaches such as vertical ridge augmentation and sinus augmentation in combination with dental implant placement. Main reasons for tooth loss are periodontal disease and dental caries, and the osseointegrated dental implant is one of the most used biomaterials to replace missing teeth with long term outcome success.^88^ The lack of supporting bone due to atrophy, trauma, failure to develop or surgical resection prevents implant placement and can be repaired via vertical ridge augmentation, illustrated in Figure 2C.^89^ Autologous bone grafting used as the BGS is considered the SOC for bone augmentation in this context.^89^ Dental implant placement in the posterior region of the maxilla is prone to implant failure caused by trauma, atrophy in the alveolar process or sinus pneumatisation, or the development of air‐filled cavities, which can be minimised by applying a sinus augmentation prior to implant placement.^90^ Sinus augmentation, illustrated in Figure 2D, enables the reduction of the sinus cavity and the filling of bone material, mostly in the form of autologous bone graft, to maximise bone area for improved implant stability.^91^

## 7 TOWARDS CLINICALLY DRIVEN ANIMAL MODELS

**FIGURE 3:**
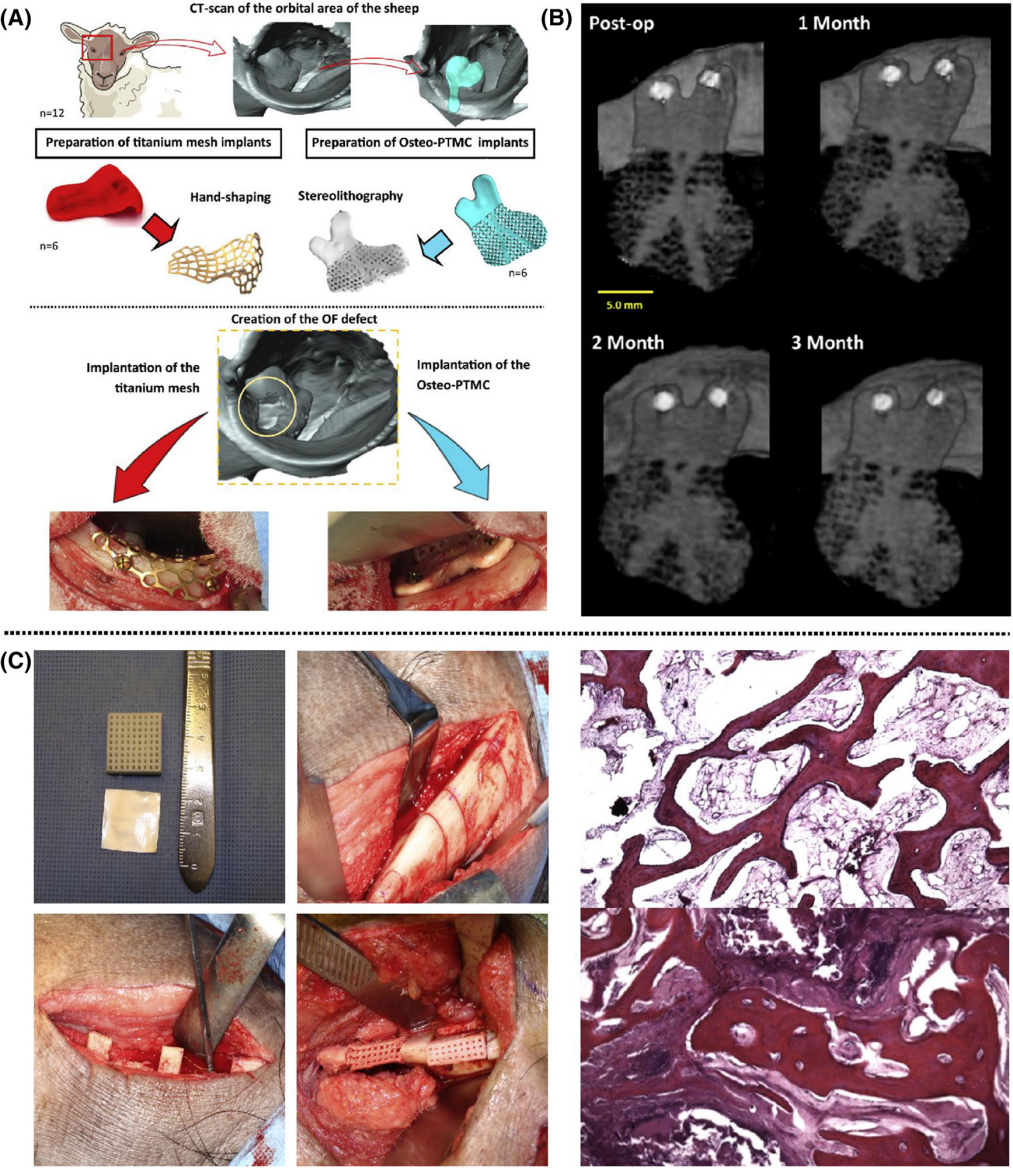
The use of large animal models for CMF application: (A) general workflow and study involving pre‐operative phase and surgery phase using an orbital floor sheep model. (B) Results of time‐lapse CT scans of the implanted 3D‐printed scaffold show increased mineralisation over time. Reproduced from Guillaume et al. with permission.^87^ (C) Left column: intraoperative images of a pig mandibular reconstruction surgery using a 3D‐printed porous scaffold. Right column: results of histology images (stained with hematoxylin and eosin) show increased bone formation in the experimental group (lower image) compared to the empty defect (upper image). Reproduced from Konopnick et al. with permission.^92^

The previous section has demonstrated that numerous studies investigating regeneration of CMF bone defects use a variety of different materials, fabrication technologies and animal models. Only 18 out of 48 studies employing a calvarial model clearly state the CMF application to be targeted with the developed material (Table 1). Conversely, 15 out of 17 studies using a mandibular model define a CMF application as their clinical target (Table 2). Only 7 out of 66 of the presented preclinical studies justify the use of a specific animal model (Table 3). Two examples of preclinical studies from Guillaume et al.^87^ and Konopnicki et al.^92^ can be highlighted in which both studies not only target a specific CMF application including employment of the appropriate defect site, but also use a large animal model with justification of its usage (Figure 3). While there are some obvious similarities in the approaches used, there remain important differences in the size/geometry of the defects, the surgical procedures, study durations and outcome assessments, which makes conclusive judgements regarding efficacy challenging, and also raises questions on the relevance of multiple animal models targeting the same CMF application. In addition, there are a number of further highly relevant clinical CMF indications that lack appropriate model systems. As an example, temporomandibular joint (TMJ) reconstruction is a particular clinical challenge, which, given the complex mechanical environment of the TMJ, poses additional concerns about how to faithfully recapitulate such an environment in a preclinical model to investigate the efficacy of regenerative approaches. Established solutions and key developments for targeting the reconstruction of TMJ have been presented in a review by Imola and Liddell^93^ and the use of preclinical models for TMJ tissue engineering has been reviewed by Almarza et al.^94^

Preclinical safety and efficacy testing of bone implants is initially performed in vitro prior to in vivo assessment.^95^ Regulatory agencies demand the validation of a preclinical animal model prior to clinical investigation, but selecting the appropriate model can be challenging.^96^ Stating the selection criteria or justification of the relevance of the chosen animal to humans is rarely included.^97^ Current ISO requirements (ISO 7405:2018) dictate that medical and dental implants should be tested in their final human form and, consequently, large animals must be employed for such pivotal preclinical efficacy testing. Thus, the choice of an appropriate experimental animal model is essential to obtain clinically justifiable preclinical data on which to base subsequent trials in humans. An animal model should guarantee minimal morbidity and maximal reproducibility but, most importantly, should faithfully reproduce the clinical condition for which the material will be employed.^4^, ^98^ From a regenerative point of view, critical size defects of CMF bones such as segmental mandibular defects pose the biggest challenge,^99^ because of their poor intrinsic healing capacity^100^ and the additional complications posed by the use of internal plate fixation in animals that may frequently fail during the time course of the study. In addition, the definition of what constitutes a critical sized defect in different species remains an important consideration in order to standardise preclinical models.^101^ This is further compounded by the additional variability also arising from the choice of specific animal species/strain, the location, size and type of defect, the choice of appropriate control groups, the time points assessed and the experimental outcome evaluation.

### 7.1 Size animal/species/strain

Small animals remain the preferred choice for most research laboratories due to the lower costs associated with animal purchase and housing, and the surgery skills are widely available.^102^ However, there are potential species‐specific differences in bone remodelling, composition and healing responses that require careful consideration to assess material efficacy between species. This is especially challenging for the CMF bone, due to the limited knowledge about the reproduction of the human condition using particular models^97^ and the lack of evidence that appendicular bone can appropriately represent CMF bone.^95^ A review on differences in large animal appendicular bone remodelling suggested human, pig, dog, sheep and goat were moderately similar, while the rabbit was least comparable.^103^ Aerssens et al. in 1998 compared the composition, density and mechanical competences of appendicular bone in human, dog, pig, cow, chicken, rat and sheep and showed distinct interspecies differences, with the dog and rat being the most and least comparable, respectively, to human bone properties.^104^ Specifically, femoral bone samples from seven species were compared and reported that rat bones differed from human bones in terms of their ash, collagen and IGF1 contents.^104^ However, studies using more modern analysis methods have challenged the relevance of these differences. A 2011 study utilising CT analysis concluded that smaller animals are a useful tool, depending on the specific research question being asked.^105^ Furthermore, other researchers provide evidence that rodent remodelling is similar to humans, thereby suggesting that rodent models are justified since the relevant cellular and molecular cues for remodelling are consistent with humans,^106^ and regulation of the process via growth factors, chemokines and cytokines is also comparable.^107^ In a 2020 study specifically investigating alveolar bone morphology, Pilawski et al. did not find evidence to conclude the superiority of pig models over rodent models in an interspecies comparison study using histology, immunohistochemistry and vital dye labels.^95^ One known biological discrepancy between rodents and humans is the reduced efficacy of rhBMP‐2 in human orofacial bone regeneration, including tooth extraction socket healing, sinus augmentation or reconstruction of alveolar clefts.^108^ Thus, this is a particularly contentious area and the limited number of in‐depth, comparative interspecies analyses, particularly in relevance to alveolar bone and CMF applications, make conclusive statements difficult. Given the fact that none of the animal models under evaluation perfectly resembles the human situation, aspects such as quantifiable differences in bone mechanical strength, size of the test material and the potential biological mechanism of action should all be considered when choosing the correct animal model.

A further issue arises concerning the predominant use of young, healthy animals in preclinical models, which does not typically reflect the increased age and potential comorbidities, such as impaired vascular function and reduced angiogenic responses,^22^ present in human patients. For example, young rabbits are often used for preclinical studies but, due to their high rate of cortical bone remodelling compared to humans, they can be a poor representative of such a process.^109^ Furthermore, aging is increasingly realised as influencing numerous cellular processes including immune responses, potentially impacting on fracture healing outcome.^110^ Until more representative preclinical models, such as those involving aged or diabetic models,^22^ the predictive nature of such studies will continue to frustrate researchers regarding clinical translation.

Studies involving the implantation of human cells use immunocompromised small animal models, thereby creating an additional issue for subsequent extrapolation to human physiology, leads to a major challenge: what is a suitable animal model to study the bone healing potential of a cell‐based therapy? It is established that immunocompetent mice have differences in bone regenerative potential compared to immunodeficient mice within the same strain.^111^ Even within species, different mice strains are known to have differences in bone mechanical properties,^112^ immune responses,^113^ rates of fracture healing^114^ and healing capacities overall.^115^ Thus, it is important to consider different strain types and include strain information in each study.^115^ Even in large animal models, immunological differences are evident, with different breeds of sheep shown to have altered disease susceptibilities,^116^ highlighting the need for this information to be routinely provided.

### 7.2 Location/size/type of defect

‘The rat calvarial defect is generally used to evaluate bone regeneration in an orthotopic model and to screen biomaterials or tissue engineering constructs before moving to larger animals for potential translation to human applications’.^77^ This is a typical statement to justify the use of a calvarial defect model after some preliminary in vitro tests. We would argue that the validation of a biomaterial/construct when tested in a single unloaded site as the calvaria does not justify efficacy in the vast field of bone regeneration, or to all CMF clinical implications. The calvarial model is a relevant model indeed, but mainly to target a cranial bone defect. Of note, the location within the cranium can also potentially alter the healing capacity, as demonstrated by the superior healing of the frontal bone compared to the parietal/temporal bones in a human calvarial study.^117^ Due to intrinsic differences between location sites (e.g. the loadbearing status of a segmental defect compared to a non‐loadbearing calvarial defect), the applicability of the results obtained with a calvarial model to any CMF site is diminished. It is known that the reconstruction of a loadbearing bone is dependent on the magnitude and frequency of loading,^118^ making a segmental loadbearing defect in an animal model clinically more relevant. So far, the clinical indication for CMF reconstruction has not been appropriately addressed, and most of the preclinical studies use predominately cylindrical defects with a 2.7–15 mm diameter in small animal models. Cylindrical defects mimic the non‐continuity mandibular defects, but a segmental defect should be used to reproduce a continuity defect. The orbital floor requires a separate investigation, even though it is not a loadbearing site, since it varies in shape, size and geometry compared to other CMF bones.^87^ Only limited studies have reported implanting tissue engineered constructs into the orbital floor.

The CMF defect models discussed in this review are the calvarial, mandibular and orbital floor defect models. In most cases, a trephine^119^, ^120^, ^121^, ^122^, ^123^, ^124^, ^125^, ^126^, ^127^, ^128^, ^129^, ^130^, ^131^, ^132^, ^133^, ^134^, ^135^, ^136^, ^137^, ^138^, ^139^, ^140^, ^141^, ^142^, ^143^, ^144^, ^145^, ^146^, ^147^, ^148^, ^149^, ^150^, ^151^, ^152^ is used to create cylindrical defects in the calvaria and the mandible. Other bone cutting devices for cylindrical defects include a circular knife,^153^ a biopsy punch,^154^ a drill^126^, ^155^, ^156^, ^157^, ^158^, ^159^ and rongeurs.^160^ The most used cutting device for creating segmental defects in the mandibular is the reciprocating bone saw, which is mainly used for large animals.^92^, ^161^ The spherical burr,^162^ diamond burr^163^ and surgical drill^164^ are also used for segmental defects in small animals. However, it is crucial that care must be taken to limit additional soft tissue damage during the procedure (e.g. the dura mater in the calvarial model) to allow effective comparisons of efficacy between groups. Hence, the bone‐cutting device should always be reported, as well as the surgical procedure applied to all the animals included in the study.

As previously stated, it is an important consideration that the chosen animal model adequately reflects the clinical problem, particularly in the CMF realm, and we encourage the use of segmental defects for the mandibular site, since they are known to be a significant issue in the CMF field. In addition, the surgical procedure should ideally be performed by the same person following extensive practice, and excluded animals should be included in the study, along with the relative reasons. To ease the comparison across studies, a standardisation of the cutting device for each animal model should also be encouraged. Detailed reporting of in vivo findings, as stipulated in the ARRIVE guidelines 2.0, should now be considered mandatory in modern publishing, to further improve reproducibility of preclinical studies.^165^

### 7.3 Control groups

In most of the reported studies mentioned here, an empty defect is used as a negative control, but only very rarely (3 out of 66 studies)^126^, ^142^, ^166^ is a positive control, such as an autologous bone graft, used as a comparator to assess ultimate efficacy of the tested material. This creates a knowledge gap in the current literature: how does the bone‐mimetic material perform in a preclinical model against the SOC? And what are the underlying biological mechanisms making bone autologous bone grafts the SOC?

To decrease costs and more importantly the number of animals used, it is common practise to create multiple defects within the same animal and, in some cases, to have control defects in close proximity to tested materials/constructs. The potential confounding effects, both local and systemic, of such an approach is difficult to assess but should be carefully considered depending on the experimental context. Indeed, the risk of systemic inflammatory responses increases during surgeries with injuries of the dura mater,^80^ or during microbial infections from intra‐oral surgical sites.^85^ Furthermore, the potential systemic effect of drug‐loaded scaffolds and the possible influence on the other defect sites should be first carefully evaluated in a pilot study.

We encourage scientists in this field to consider the effect of local and systemic inflammatory responses to the experimental outcome but, more importantly, to implement a positive control such as the autologous bone graft, in addition to empty defect controls, in future studies.

### 7.4 Study duration including time points and analysis strategy

In the presented studies, the endpoint and time points appear to follow an overall trend, with typical endpoints ranging from 8–12 weeks, which resembles the typical bone healing process of 6–8 weeks (and in some cases, 12 weeks) in humans and also to make use of appropriate endpoints to validate the outcome. The size of the animal model has an impact on the additional intermediate time points as demonstrated by the fact that 1 to 4 additional time points are included for small animal models while for large animal models typically only include 1 or 2 time points, likely due to the increased costs associated with large animal studies.

Unlike histology and immunohistochemistry analyses, which require euthanasia of the animal, radiographical imaging such as 3D image acquisition (CT, μCT) or 2D radiographs can be used to longitudinally assess bone healing in the same animal over time, which is an attractive means to reduce animal usage. Nevertheless, histology remains the main analysis method, and it is used in all presented studies. Histology is a powerful tool to assess the infiltration of native tissue within the construct, which makes it one of the most important outcome assessments. This is closely followed by CT/μCT (55 out of 66 studies), immunohistochemistry (16 out of 66 studies) and 2D radiography (3 out of 66 studies). Mechanical testing of regenerated areas is also used as an evaluation strategy (6 out of 66). To quantify the amount of repaired bone from histological and/or immunohistochemical analysis, either image analysis software is used (mostly ImageJ,^92^, ^125^, ^127^, ^132^, ^142^, ^143^, ^150^, ^161^, ^167^, ^168^, ^169^ Image‐Pro Plus^119^, ^122^, ^157^, ^170^, ^171^, ^172^ or i‐solution^120^, ^133^, ^136^), or a scoring system^173^ is applied. Approximately 50% of studies do not show quantification of the histological and immunohistochemical images leading to potential biased and subjective analyses. New bone regeneration quantified from radiographical imaging is mostly expressed in the form of ‘bone volume/total volume (BV/TV)’, bone mineral density, new bone formation or Hounsfield Units. A variety of software are used to quantify radiographical images including Nrecon,^136^, ^139^, ^140^, ^148^, ^150^, ^151^, ^169^, ^174^ Amira,^87^, ^142^, ^152^ Skyscan,^123^, ^137^, ^164^ AsanJ‐Morphometry software,^138^ InVesalius 3^166^ and many more.

**FIGURE 4:**
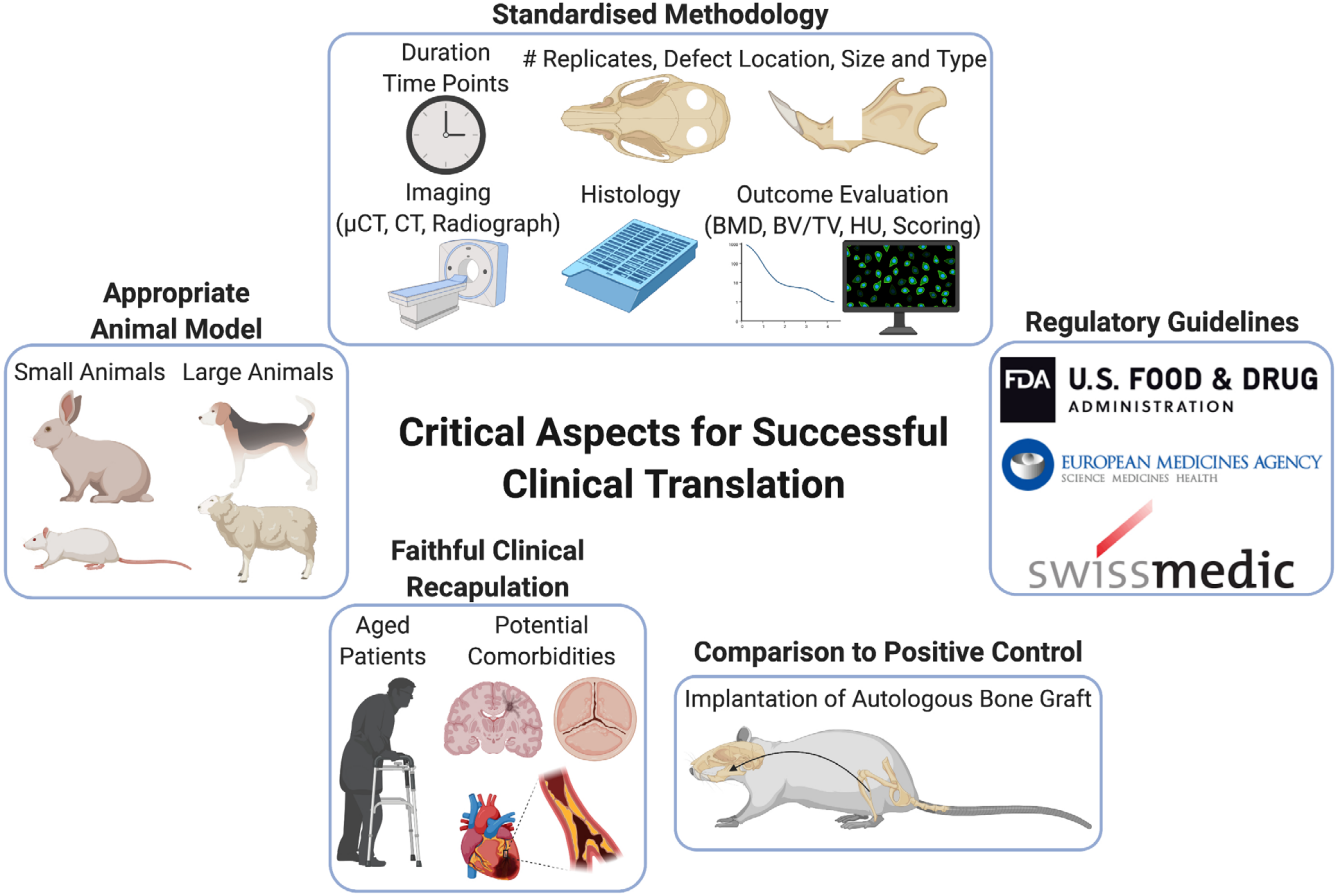
Towards clinically driven animal models: suggested guidelines. uCT: micro‐computed tomography, BMD: bone mineral density, BV/TV: bone volume/total volume; HU: hounsfield units. Created with BioRender.com

To improve consistency across studies, we would strongly encourage that the study endpoint, time points and analytical methods be standardised based on individual species. We strongly suggest to at least include histology to assess the native tissue infiltration capacity, as well as CT scanning to measure the newly formed bone volume (BV/TV) in any pre‐clinical study. The parameter outcome of BV/TV measurements based on CT scanning is the most important outcome evaluation, because the clinical evaluation of newly formed bone is also based on CT scanning, and it is therefore highly recommended to be included in the preclinical study. Additional assessments such as immunohistochemistry or mechanical testing are welcome additions. The minimum recommended number of timepoints are two, the first time point being after 4 weeks, to assess the performance of bone repair during the earlier stage of the healing process, and the second time point after 8 weeks when the healing process is typically viewed as sufficient to adequately withstand mechanical loading etc. (with the caveat that additional longer‐term studies would be required to assess the remodelling process and ultimate integration of the construct, where this is applicable). More timepoints are encouraged only if necessary, to prevent unnecessary use of experimental animals. Summarised suggested guidelines to improve the use of clinically driven animal models is shown as a schematic overview in Figure 4.

### 7.5 Selection of the material

There is a large selection of possible materials and combinations to choose from that ranges from natural polymers such as collagen, gelatin, silk or alginate; synthetic polymers such as PLGA, poly(propylene fumarate) and PCL; bioceramics such as HAp, TCP and bioactive glass; biodegradable metals such as magnesium and its alloys; and carbon‐based nanomaterials such as carbon nanotubes and graphene.^175^ To name a few novel combinations: PCL functionalised with deferoxamine,^176^ magnesium incorporated into a PLGA/TCP scaffold,^177^ mesoporous bioactive glass for the delivery of growth factors^178^ and chemically synthesised phosphate graphene.^179^ However, these materials require further evaluation for efficacy in CMF‐specific contexts. A cyclic pathway on how to design a material for bone tissue engineering, specifically in terms of strategy, optimisation cycle and evaluation is proposed in a review by Koons et al., which also presents recent advances and development strategies in this field.^175^ Advances in tissue‐engineered bone technology and future aspects are also discussed in a review by Tang et al.^180^

The choice of the material must be based on the application. In this review, the focus lays in 3D‐printed scaffolds for CMF application. Due to non‐loadbearing nature of the calvarial defect, a suitable material does not require to have high stiffness and resilience. Conversely, these properties might be essential for materials used to regenerate loadbearing segmental mandibular defects. We have previously highlighted the importance of material properties and vascularisation for a successful initial interaction with the host tissue. The implanted construct should lead to an early invasion of immune cells, bone cells, progenitor cells and vascular cells. To test the native ability of a material for integration with the host tissue, it should be additionally assessed in the absence of cell encapsulation.

**FIGURE 5:**
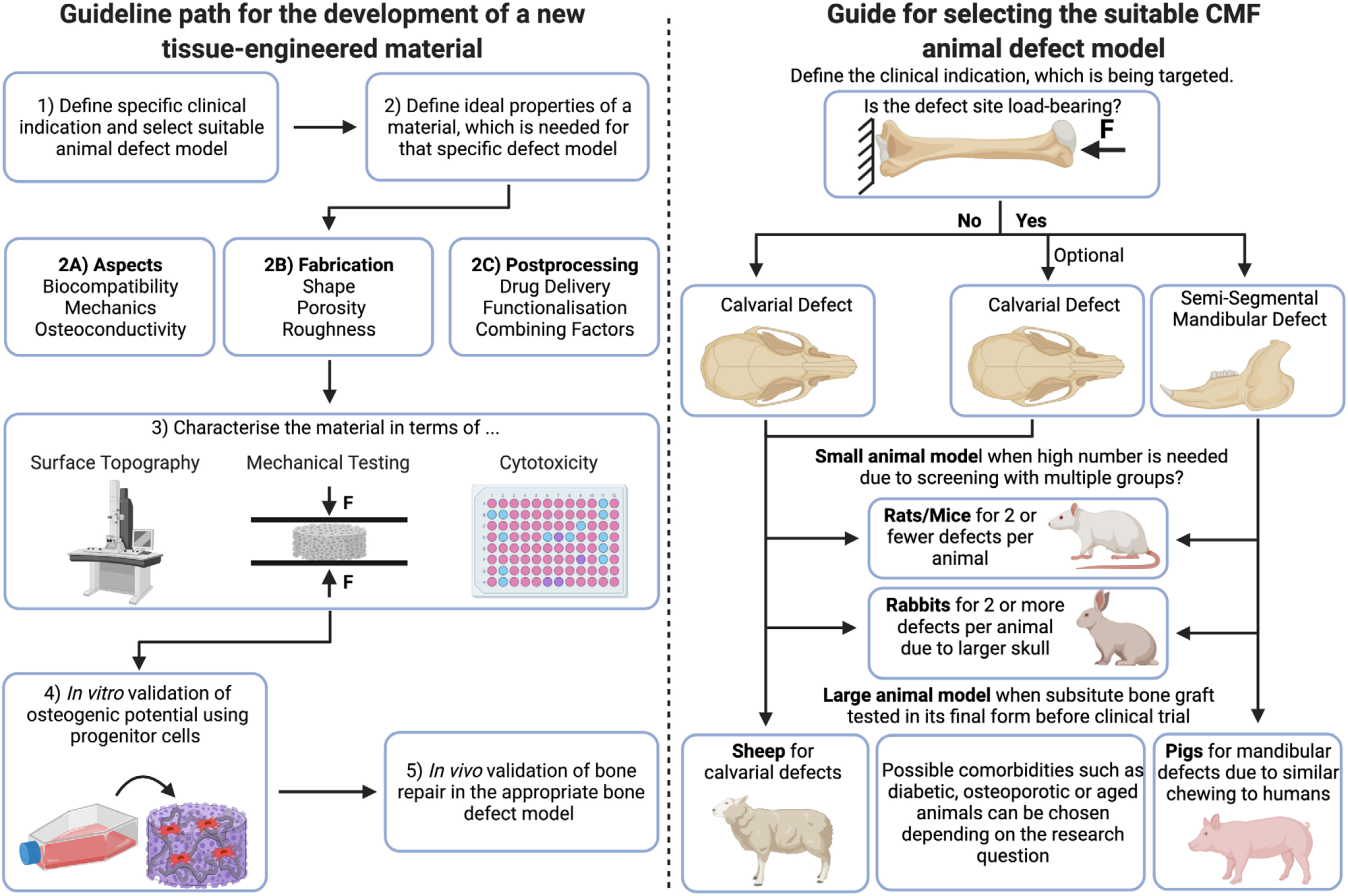
Left schematic: guideline path for the development of a new tissue‐engineered material repair purposes. Right schematic: guide for selecting the suitable CMF animal defect model. Created with BioRender.com

We propose a clinically driven guideline path for the development of a new TE material for CMF repair purposes, as well as guidelines for selecting the suitable CMF animal defect model (Figure 5).

**FIGURE 6:**
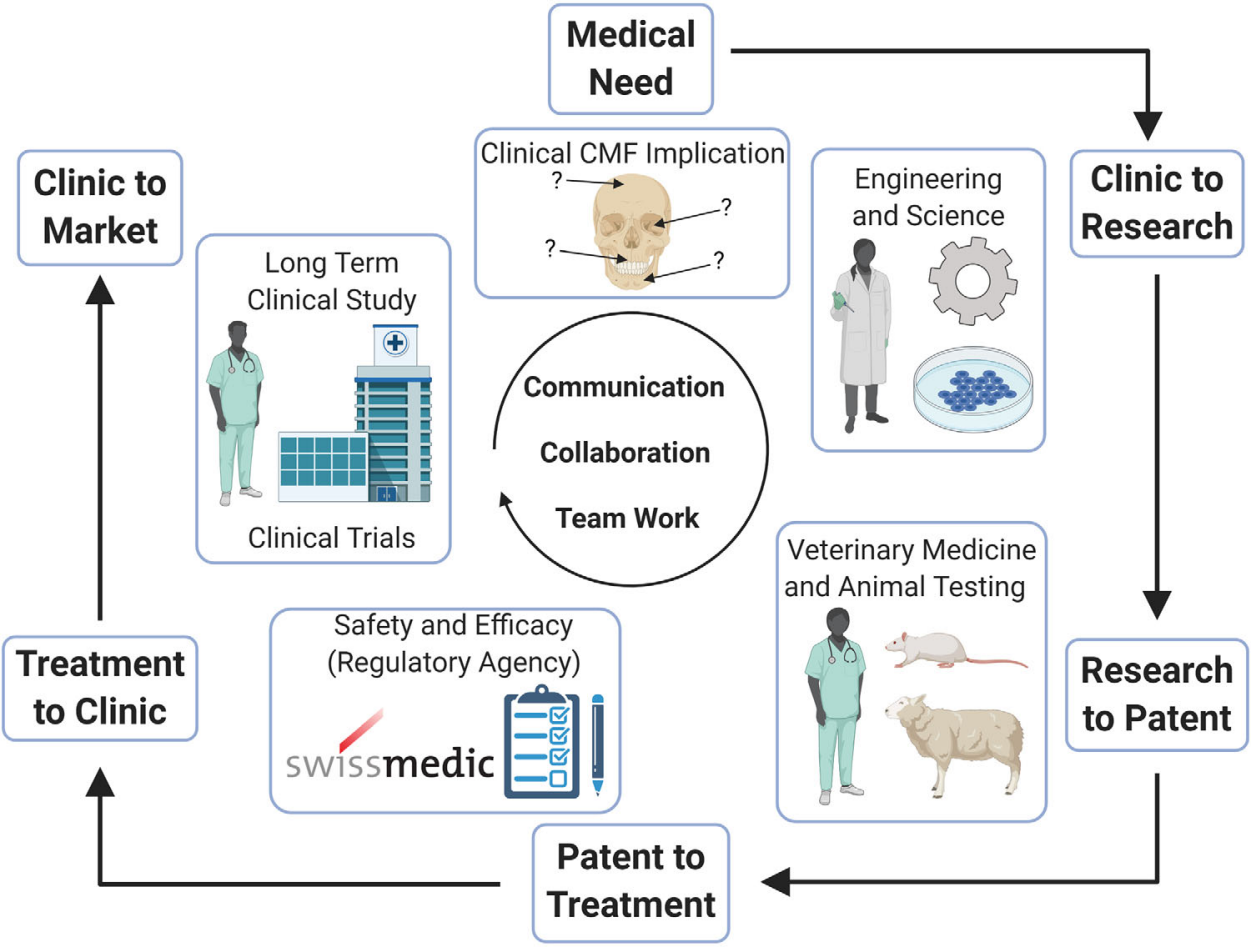
Ideal translation based on a multidisciplinary approach. Created with BioRender.com

The clinical translation of a TE material requires a step‐by‐step approach that starts from a medical need and ultimately ends with a product on the market. Its success depends on clear communication, constant collaboration and teamwork across multidisciplinary expertise (Figure 6). Without such an approach, we fear that the field of bone tissue engineering may continue to frustrate, with a continued lack of viable BGS for replacement of autografts. Indeed, a search via ‘ClinicalTrials.gov’ using the search terms ‘3D printing, 3D‐printed bone graft, substitute, or scaffold’ for the efficacy testing of 3D‐printed BGS in patients demonstrates that only a limited number of 3D‐printed constructs have entered early clinical trials, with no published findings to date.

## 8 CONCLUSIONS

Tissue engineering has the potential to revolutionise the field of CMF bone regeneration but, so far, the implementation of promising materials/constructs into the clinic has been very limited. The provision of scientific evidence justifying the clinical translation of a tissue engineering product is a major undertaking and, in this respect, preclinical animal models are a critical resource necessary to test safety and efficacy. Although an experimental preclinical model cannot fully replicate the human disease, we should aim to maximise the quality of the experimental data generated to increase the translation potential of the material in question. With this aim in mind, the clinical scenario should be used as main driver of the choice of the model and a rigorous scientific rationale should be applied, to justify the decision. The challenging nature of bone reparative approaches, requiring a thorough appreciation of both biological and mechanical processes involved, requires a multidisciplinary approach. Improvements to standardised assessment protocols across studies is encouraged, as well as sharing the knowledge and experiences of engineers, scientists, veterinarians and CMF surgeons, to ultimately establish a series of robust guidelines supporting the development of a new tissue engineered material and to facilitate comparisons between results from different research groups.

## CONFLICT OF INTEREST

The authors declare no conflict of interest.
